# The Inhibition of Ureteral Motility by Periureteral Adipose Tissue

**DOI:** 10.5402/2012/312487

**Published:** 2012-03-07

**Authors:** Lyndsey M. Killian, Stuart J. Bund

**Affiliations:** Health Sciences Centre, UCD School of Medicine and Medical Science, University College Dublin, Belfield, Dublin 4, Ireland

## Abstract

Perivascular adipose tissue exerts an anticontractile influence on vascular smooth muscle. This study was conducted to determine whether periureteral adipose tissue (PUAT) could exert a similar influence upon ureteral smooth muscle. Acetylcholine-stimulated (10^−7^ M–10^−4^ M) contractile responses of ureteral segments obtained from male Wistar rats were recorded in the presence and absence of PUAT. Ureters with PUAT generated phasic contractile responses with significantly lower frequencies (*P* < 0.001) and magnitudes (*P* < 0.001) compared with ureters cleared of their periureteral adipose tissue. Removal of PUAT significantly increased the frequency (*P* < 0.01) and magnitude (*P* < 0.01) of the contractile responses. Bioassay experiments demonstrated that ureters with PUAT released a transferable factor that significantly reduced frequencies (*P* < 0.05), but not magnitudes, of the contractile responses of ureters cleared of PUAT. The nitric oxide synthase inhibitor L-NNA (10^−4^ M) did not significantly influence the anticontractile effect exerted by ureters with PUAT. This is the first study to demonstrate that ureteral motility is influenced by its surrounding adipose tissue. The PUAT has an anticontractile effect which is mediated by a transferable factor released from the PUAT. The identity of the factor is unknown but does not exert its effect through nitric oxide.

## 1. Introduction

Adipose tissue was once recognised as only having protective and thermal functions, but it is now characterised as an endocrine organ mediating its paracrine effects on neighbouring tissues through lipid and protein mediators known as adipokines [1]. It has recently been demonstrated that vascular smooth muscle is influenced by its surrounding adipose tissue. Specifically, the perivascular adipose tissue (PVAT) exerts an anticontractile effect on the vasculature. Rat mesenteric arteries [2, 3] and human subcutaneous arteries [4] with intact PVAT display diminished contractile responses in comparison with those cleared of PVAT. Similar results have been observed in rat aortic ring preparations where it was identified that a transferable factor originating from the adipose tissue is responsible for the effect [5]. The transferable factor mediates its effects in an endothelium-dependent and- independent fashion [5]. Cultured endothelial cells generate nitric oxide (NO) in response to medium conditioned by PVAT [5] and NOS inhibition attenuates the anticontractile activity [4]. Adiponectin has emerged as a candidate for the PVAT-derived anticontractile factor [4, 6]. Unlike other adipokines, adiponectin expression is restricted to the adipocyte cells of adipose tissue [7].

To date, investigations into the anticontractile effect of adipose tissue have been confined to vascular smooth muscle preparations. In the present study the question of whether the periureteral adipose tissue (PUAT) exerts an anticontractile effect on ureteral smooth muscle, in a similar fashion to the effect of PVAT on vascular smooth muscle, has been addressed. The study was therefore designed to test the hypothesis that PUAT attenuates ureteral motility.

## 2. Methods

Male Wistar rats with body weights ranging from 241 to 531 g were used in this study. They were housed at the Biomedical Facility, University College Dublin, where they were maintained on standard rat chow and tap water ad libitum. All procedures were performed in accordance with University College Dublin institutional guidelines.

Rats were humanely killed by stunning and cervical dislocation. Both ureters were removed immediately and placed into a petri dish containing PSS of the following composition (mM): NaCl 119, KCl 4.7, NaHCO_3_ 25, KH_2_PO_4_ 1.18, MgSO_4_ 1.17, K_2_-EDTA 0.026, D(+)-glucose 5.5, and CaCl_2_ 1.6. Each ureter was noted as being either left or right and tissue preparation commenced immediately. Under a binocular microscope the surrounding adipose tissue was carefully dissected free or it was left intact as required. Segments of ureter 15 mm in length were then prepared for assessment of contractile function. Ureteral segments were suspended in 20 mL capacity water-jacketed tissue baths containing PSS: one end was secured to a tissue holder in the bath by means of cotton thread while the other was secured to a force transducer in a similar fashion. The baths were then warmed to 37°C and gassed with 5% CO_2_ in air. A pretension of 0.2 g was applied to facilitate maximal contractile responses in this preparation (unpublished observations). Ureteral segments were allowed to equilibrate under these conditions for 40 minutes before the addition of any reagents.

### 2.1. Chemicals and Reagents

All chemicals and reagents were obtained from Lennox Laboratory Supplies (Dublin, Ireland). ACh was dissolved in deionised water (10^−2^ M), divided into 1 mL aliquots, and then stored frozen (−20°C) until required. Serial dilutions of the 10^−2^ M ACh stock solution were made in PSS when required. L-NNA (10^−2^ M) was prepared fresh each day as required in PSS.

### 2.2. Protocol 1: Influence of Periureteral Adipose Tissue

In this protocol ureteral segments were used with PUAT intact (*n* = 12) or dissected free (*n* = 12), with left or right segments (both taken from the same rat) subject to PUAT removal in alternate fashion. Each segment was suspended in a separate tissue bath and force recordings were made simultaneously. After the equilibration period acetylcholine (ACh, 10^−7^−10^−4^ M) was applied in a cumulative fashion. Each concentration was applied for four minutes. ACh was the only agonist used in this and all subsequent protocols because it consistently stimulates contractile responses; unpublished observations in our laboratory have demonstrated that adrenoceptor agonists such as noradrenaline and phenylephrine do not.

### 2.3. Protocol 2: Bioassay

This protocol was conducted immediately after the washout at the end of protocol 1 and utilised half of the PUAT-free ureters tested in protocol 1. The ureter with PUAT was removed from its bath and then loosely suspended in the tissue bath that contained the contralateral PUAT-free ureter and a further equilibration for 40 minutes ensued. ACh-stimulated contractions of the cleaned ureter were then recorded with an identical ACh application protocol as described above.

### 2.4. Protocol 3: Time Control

The remaining clean ureters (*n* = 6) following protocol 1 were used. Following washout of the ACh a second 40 minute period elapsed prior to a repeat of the ACh challenge.

### 2.5. Protocol 4: PUAT Removal

This protocol was applied to PUAT intact ureters subsequent to completion of protocol 1 (*n* = 4) and to PUAT intact ureters following completion of protocol 2 (*n* = 4). The ureter was removed from the tissue bath and placed in a petri dish containing PSS. The surrounding adipose tissue was dissected free from the ureter under a binocular microscope and both ends of the ureter were retied with thread. The ureter was resuspended in the bath for a further 40 minutes equilibration period. ACh was applied in the same manner as described above for protocol 1 and was therefore either the second or third challenge with ACh.

### 2.6. Protocol 5

PUAT-free ureteral segments (*n* = 10) were suspended in a tissue bath and challenged with ACh in an identical fashion to that of protocol 1. The contralateral ureter with PUAT intact was then loosely suspended in the same tissue bath as the PUAT-free ureteral segment. The nitric oxide synthase inhibitor L-NNA (10^−4^ M) was applied to the bath and, following a further 40 minute period the ACh challenge was repeated. Left and right ureters were used as PUAT-free segments in alternate fashion.

## 3. Data Analysis

ACh responses were recorded using the AcqKnowledge data acquisition software. Contractile responses were phasic in nature and the responses in the last three minutes (i.e., stable responses) of each ACh application were used for analysis purposes. Frequencies (min^−1^) and magnitudes (g) were analysed. Concentration response curves were analysed by means of repeated measures analysis of variance (RMANOVA) using the SPSS statistical program. When statistically significant differences were detected, the responses at each concentration were compared between groups by means of paired *t*-tests modified by the false discovery rate procedure [8] to cater for multiple comparisons. Data are presented as mean ± SEM throughout and differences between groups were considered statistically significant when *P* < 0.05.

## 4. Results

**Figure 1:**
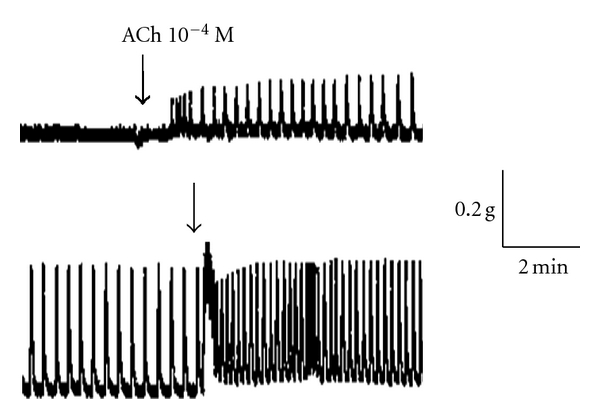
Sample recording of Ach-induced ureteral contraction. ACh-induced contractile responses in a ureter with PUAT removed (lower trace) were greater than those of the contralateral ureter + PUAT (upper trace). ACh (10^−5^ M) was present and increased to 10^−4^ M at the time point indicated.

**Figure 2:**
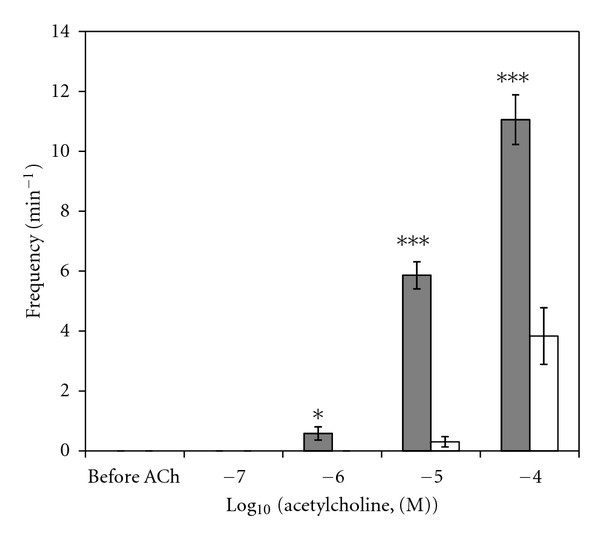
Ureteral contractile responses to ACh in the presence and absence of PUAT. Ureters + PUAT (open bars, *n* = 12) and ureters with PUAT removed (closed bars, *n* = 12). Frequencies were significantly attenuated in the ureter + PUAT group (*P* < 0.001 RMANOVA group term and group × concentration term). *P < 0.05, ***P < 0.001.

**Figure 3:**
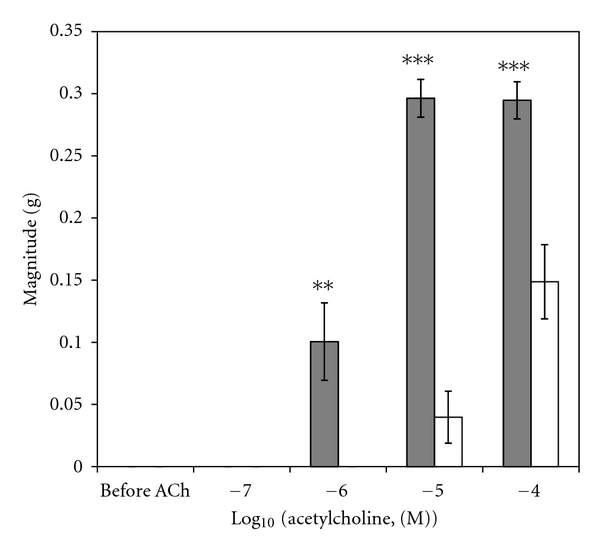
Ureteral contractile responses to ACh in the presence and absence of PUAT. Ureters + PUAT (open bars, *n* = 12) and ureters with PUAT removed (closed bars, *n* = 12). Magnitudes were significantly attenuated in the ureter + PUAT group (*P* < 0.001 RMANOVA group term and group × concentration term). **P < 0.01, ***P < 0.001.

**Figure 4:**
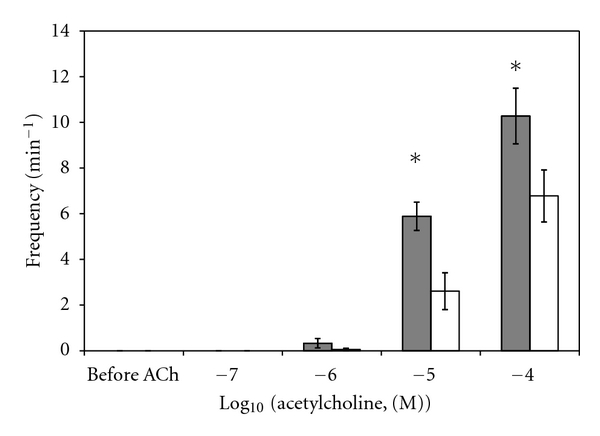
Influence of coincubation with PUAT on contractile responses to ACh on PUAT-free ureters. Frequency of contractile responses to ACh of ureters with PUAT removed in the absence (closed bars, *n* = 6) and presence (open bars, *n* = 6) of ureters with PUAT. Frequencies were significantly attenuated in the presence of a ureter + PUAT (*P* < 0.05 RMANOVA group term; group × concentration term, *P* = 0.07). *P < 0.05.

**Figure 5:**
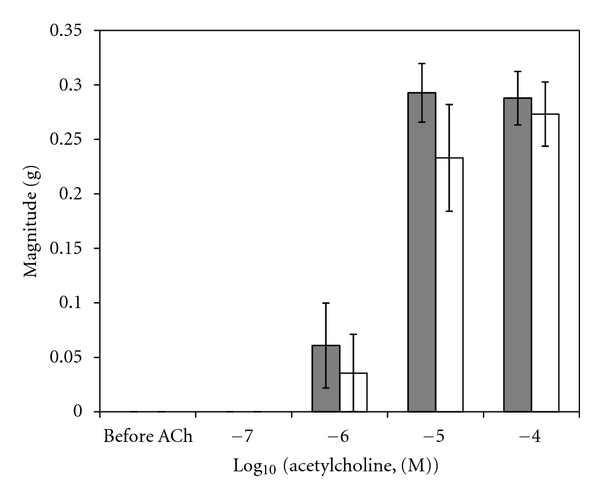
Influence of coincubation with PUAT on contractile responses to ACh on PUAT-free ureters. Magnitude of contractile responses to ACh of ureters with PUAT removed in the absence (closed bars, *n* = 6) and presence (open bars, *n* = 6) of ureters with PUAT. Magnitudes were not significantly different.

A sample original recording obtained in protocol 1 is shown in Figure 1. ACh-stimulated contractile responses of PUAT intact ureteral segments were significantly reduced compared to those of PUAT-free ureters in terms of both frequency (Figure 2) and magnitude (Figure 3). After equilibration in the presence of a ureter with intact PUAT (protocol 2), the frequency of the PUAT-free ureteral contractions were significantly reduced (Figure 4). Amplitudes of contraction were also reduced but the effect was not statistically significant (Figure 5).

**Figure 6:**
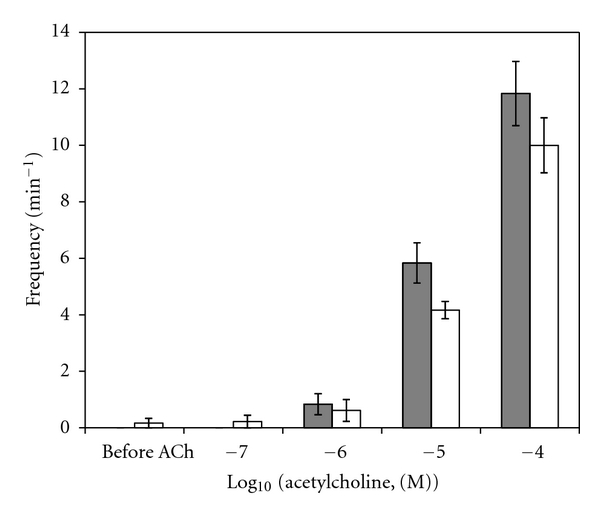
Influence of repetitive application of ACh on PUAT-free ureteral contractile responses. There were no significant differences in frequency between the first ACh challenge (closed bars, *n* = 6) and the second ACh challenge (open bars, *n* = 6).

**Figure 7:**
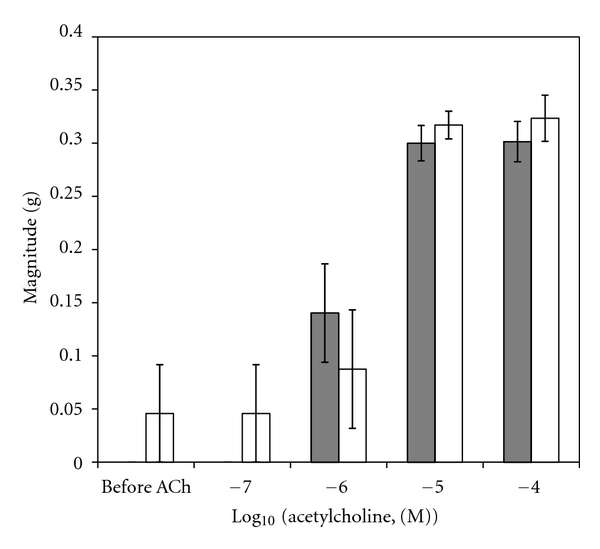
Influence of repetitive application of ACh on PUAT-free ureteral contractile responses. There were no significant differences in magnitudes between the first ACh challenge (closed bars, *n* = 6) and the second ACh challenge (open bars, *n* = 6).

Time control experiments (protocol 3) revealed that ACh concentration response relationships were not significantly different when two ACh challenges were separated by 40 minutes (Figures 6 and 7).

**Figure 8:**
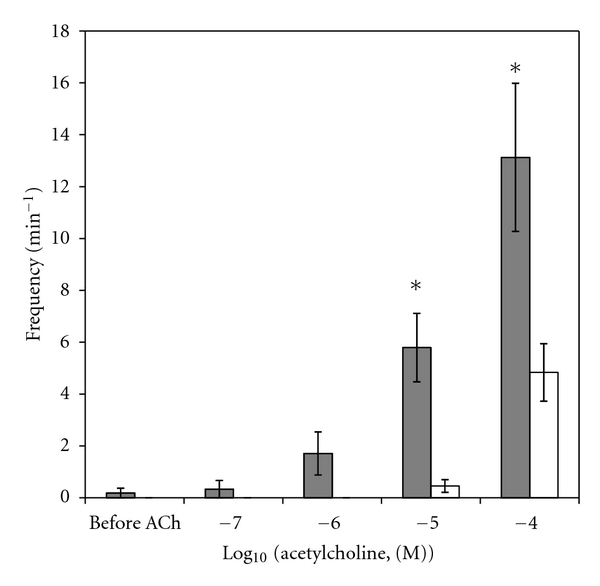
Influence of removal of PUAT on ureteral contractile responses to ACh. Contraction frequencies were significantly increased (RMANOVA *P* < 0.01 group term, *P* < 0.05 group × concentration term) in the ureters following PUAT removal (closed bars, *n* = 8) compared to those prior to PUAT removal (open bars, *n* = 8). *P < 0.05.

**Figure 9:**
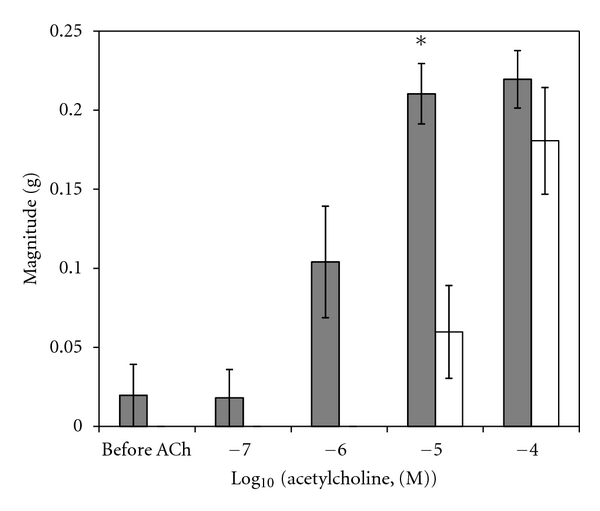
Influence of removal of PUAT on ureteral contractile responses to ACh. Contraction magnitudes were significantly increased (RMANOVA *P* < 0.01 group term, *P* < 0.05 group × concentration term) in the ureters following PUAT removal (closed bars, *n* = 8) compared to those prior to PUAT removal (open bars, *n* = 8). *P < 0.05.

**Figure 10:**
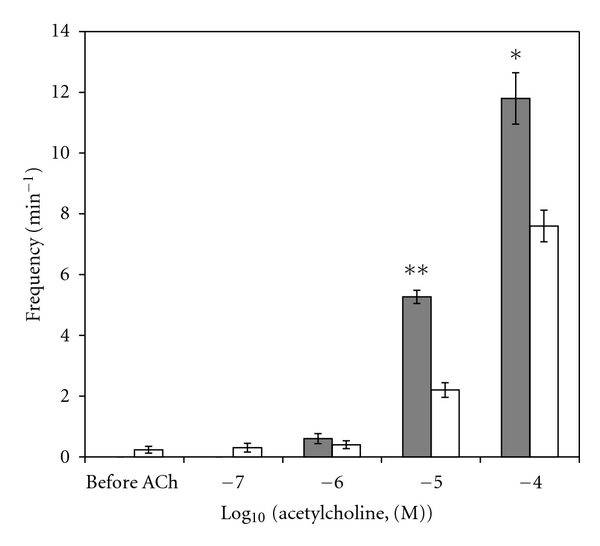
Influence of coincubation with PUAT + L-NNA on contractile responses to ACh of PUAT-free ureters. ACh stimulated contractile frequencies of PUAT-free ureters remained significantly greater in the absence (control, closed bars, *n* = 10) than those in the presence (open bars, *n* = 10) of a ureter + PUAT and L-NNA. (RMANOVA *P* < 0.05 group term, *P* < 0.001 group × concentration term). *P < 0.05, **P < 0.01.

**Figure 11:**
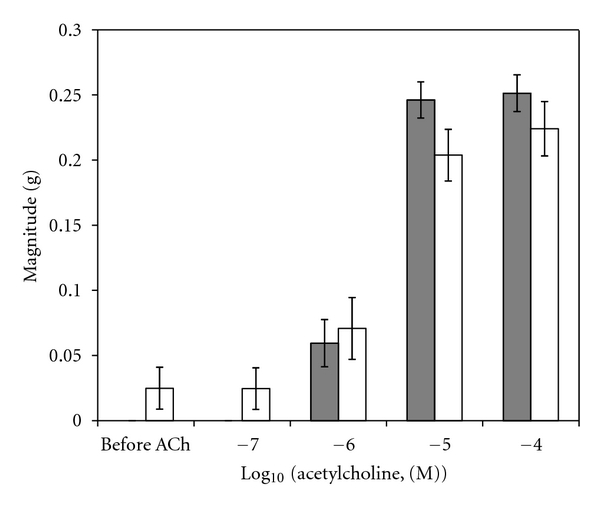
Influence of coincubation with PUAT + L-NNA on contractile responses to ACh of PUAT-free ureters. ACh stimulated contractile magnitudes of PUAT-free ureters in the absence (control, closed bars, *n* = 10) and presence (open bars, *n* = 10) of a ureter + PUAT and L-NNA. Contractile magnitudes were not significantly different.

Removal of PUAT from ureteral segments (protocol 4) significantly increased the frequency (Figure 8) and magnitude (Figure 9) of contractile responses in all cases. In the presence of L-NNA (protocol 5) the presence of PUAT remained associated with reduced contractile frequencies (Figure 10). Consistent with no significant reduction in contractile magnitude in the presence of PUAT in the bioassay experiments (Figure 5), contractile magnitudes of PUAT-free ureters were also not significantly affected by the presence of PUAT plus L-NNA (Figure 11).

## 5. Discussion

The present study provides evidence for an adipose tissue derived anticontractile effect on ureteral motility. We have demonstrated that periureteral adipose tissue (PUAT) has an anticontractile effect on ureteral smooth muscle which is mediated by a transferable factor. The identity of that factor is not known but it operates in a nitric oxide-independent fashion to reduce the frequency of ureteral phasic contractions. We could not demonstrate that the factor acts in a transferable fashion to diminish the magnitude of contractile responses but removal of PUAT significantly enhanced the magnitude demonstrating that it has the capacity to at least act in a paracrine fashion. Accordingly, PUAT negatively diminishes smooth muscle contractile function in a manner similar to that described for perivascular adipose tissue [2–5].

The transferable factor originating from perivascular adipose tissue has been shown to mediate its anticontractile effects by endothelium-dependent and independent means [5]. The endothelium-dependent relaxation mechanism involves the release of an adipose derived relaxing factor (ADRF) resulting in increased NO production in the endothelium with subsequent K^+^ channel activation and relaxation [5]. Voltage- [3] and Ca^2+^-dependent [5, 9] K^+^ channels have been implicated in this regard. The endothelium-independent relaxation mechanism involves the generation of H_2_O_2_ within the adipose tissue with subsequent activation of soluble guanylyl cyclase and relaxation of the smooth muscle [5].

Adiponectin has been identified as the ADRF in human small arteries, with the anticontractile effect inhibited by adiponectin type-1 receptor blocking peptide and the nitric oxide synthase inhibitor L-NMMA [4]. In support of this identification adiponectin increases NO production in bovine aortic endothelial cells [6] and exogenous application of adiponectin to rat mesenteric arteries results in a vasodilatation [4]. Furthermore, adiponectin administration ameliorates the hypertension exhibited by obese mice [10].

The mechanism by which PUAT mediates its anticontractile effects has not been identified. The urothelium of rats has been shown to generate NO [11] and L-NNA has been demonstrated to inhibit porcine ureteral relaxations which can be reversed by L-arginine [12]. Therefore, attempts were made to determine whether NO mediates the anticontractile effect of PUAT in the present study. The application of L-NNA was without effect on ureteral contractile responses; it did not prevent the reduction of contractile frequency exerted by PUAT in bioassay experiments. This suggests that the PUAT anticontractile effect is not mediated by NO. PUAT did not reduce the contractile magnitude in bioassay experiments but the presence of an intact PUAT layer was associated with a reduction in the contractile magnitude. The reasons for this discrepancy remain unclear. It is possible that there was a higher local concentration of PUAT-derived ADRF when it acted in a paracrine fashion upon ureteral smooth muscle with an intact PUAT layer, while at the lower concentrations expected in the bioassay medium it has the capacity to diminish the frequency and not the magnitude of the contractile response. Given that PUAT in the bioassay experiments did not diminish the magnitude of the contractile responses of PUAT-free ureters the lack of an inhibitory effect exerted by L-NNA upon magnitude is not surprising. The absence of an influence of L-NNA upon ureteral contractile responses in this study is consistent with previous unpublished observations from our laboratory.

The apparent difference in the anticontractile mechanisms in the ureter and the vasculature may be due to regional differences in adipose tissue function. It has been suggested that adipose depots in different anatomical locations vary in function; preadipocyte cell characteristics of mesenteric adipose tissue differ to those of omental adipose tissue in several respects [13]. It has also been reported that there are regional differences in adiponectin secretion in human adipose tissue [14]. This latter observation may explain the difference in the anticontractile mechanism observed in the ureter compared to the vasculature but, as yet, any suggested mechanism remains speculative. Adipose tissue expresses a range of different mediators such as adrenomedullin, resistin, visfatin, leptin, IL-6, IL-8, and MCP-1 [15], any of which could be a possible mediator of this anticontractile effect in the ureter. A further germane consideration is the mode of contraction; ureteral smooth muscle exhibits phasic contractile behaviour while that of vascular smooth muscle is tonic in nature. Therefore the possibility of different sites of action for ADRF in different smooth muscle arises.

Recent studies on vascular smooth muscle have described other possible anticontractile mediators released from adipose tissue. Angiotensin-(1–7) (Ang-1–7) was shown to be in abundance in PVAT and has a clear anticontractile effect on rat aorta which was susceptible to NOS inhibition [9]. Ang-(1–7) binds to Mas receptors on endothelial cells resulting in the activation of endothelial NOS (eNOS) [16]. The attenuation of contractions by Ang-(1–7) was demonstrated in control mice but was absent in Mas receptor knockout mice [17]. Palmitic acid methyl ester has also been described as an ADRF [18].

The influence of adipose tissue on vascular smooth muscle has been shown to be important in some pathophysiological conditions. The anticontractile effect is decreased in aortic preparations of spontaneously hypertensive rats (SHR) [18, 19] and in hypoxia [4], increased in the aorta of hyperglycemic rats [20], and abolished in human small arteries in obesity [4]. Furthermore, nicotine exposure may attenuate the anticontractile effect [21]. The amount of adipose tissue surrounding the smooth muscle may also be of importance [3, 22]. The physiological role of PUAT remains to be established but it may be hypothesised that PUAT has a role in the maintenance of ureter patency, especially during times of increased intra-abdominal pressure. A role for PUAT in the regulation of ureteral peristaltic rhythm in vivo remains to be identified. However, it would be interesting to determine whether abnormalities of PUAT anticontractile effects account for abnormal ureteric motility such as that of vesicoureteral reflux.

In summary, the results from the present study demonstrate for the first time that PUAT negatively influences ureteral motility. The anticontractile effect is mediated by a transferable factor that remains to be identified but its activity is not accounted for by a nitric oxide dependent mechanism.
